# Macrophages Promote Ovarian Cancer-Mesothelial Cell Adhesion by Upregulation of ITGA2 and VEGFC in Mesothelial Cells

**DOI:** 10.3390/cells12030384

**Published:** 2023-01-20

**Authors:** Seung-Kye Cho, Kijun Lee, Jeong-Hwa Woo, Jung-Hye Choi

**Affiliations:** 1 Department of Biomedical and Pharmaceutical Sciences, Kyung Hee University, Seoul 02447, Republic of Korea; 2 Division of Molecular Biology, College of Pharmacy, Kyung Hee University, Seoul 02447, Republic of Korea

**Keywords:** mesothelial cells, macrophage, ovarian cancer, adhesion, ITGA2, VEGFC

## Abstract

Ovarian cancer is a metastatic disease that frequently exhibits extensive peritoneal dissemination. Recent studies have revealed that noncancerous cells inside the tumor microenvironment, such as macrophages and mesothelial cells, may play a role in ovarian cancer metastasis. In this study, we found that human ovarian cancer cells (A2780 and SKOV3) adhered more to human mesothelial Met5A cells stimulated by macrophages (M-Met5A) in comparison to unstimulated control Met5A cells. The mRNA sequencing revealed that 94 adhesion-related genes, including *FMN1, ITGA2*, *COL13A1*, *VEGFC*, and *NRG1,* were markedly upregulated in M-Met5A cells. Knockdown of ITGA2 and VEGFC in M-Met5A cells significantly inhibited the adhesion of ovarian cancer cells. Inhibition of the JNK and Akt signaling pathways suppressed ITGA2 and VEGFC expression in M-Met5A cells as well as ovarian cancer-mesothelial cell adhesion. Furthermore, increased production of CC chemokine ligand 2 (CCL2) and CCL5 by macrophages elevated ovarian cancer-mesothelial cell adhesion. These findings imply that macrophages may play a significant role in ovarian cancer-mesothelial cell adhesion by inducing the mesothelial expression of adhesion-related genes via the JNK and Akt pathways.

## 1. Introduction

Ovarian cancer is the most lethal gynecological cancer and a leading cause of cancer-related death in women [1]. This fatal outcome is mostly attributed to the metastatic spread of cancer cells within the peritoneal cavity. One of the characteristics of ovarian cancer cells is dissemination into peritoneal sites, such as the uterus, hepatic hilum, spleen, and omentum. The peritoneal mesothelium, which lines the peritoneal cavity and internal organs, is made up of an extensive monolayer of mesothelial cells [2]. Notably, the mesothelium is the site where the ovarian cancer cells mainly spread, and adhesion to mesothelial cells is considered a key step in the metastasis of ovarian cancer cells [3].

Emerging evidence suggests that the microenvironment of tumors, as well as their inherent characteristics, might influence the development of many malignancies, including ovarian cancer [4,5]. The tumor microenvironment comprises not only malignant cells but also many other non-malignant cell types. Among the variety of noncancerous cells in the tumor microenvironment [6,7], macrophages, more specifically known as tumor-associated macrophages (TAMs), have attracted attention because of their potential pro-tumor activities in various types of cancers, including ovarian cancer. Macrophage density has a significant positive correlation with the stage and grade of ovarian cancer [8]. High levels of TAMs in tumor tissues have been suggested to predict a poor prognosis of ovarian cancer [9,10]. Tissue-resident macrophages in the omentum have been suggested to promote the peritoneal spread of ovarian cancer cells [11]. Additionally, TAMs are associated with clinically relevant immunosuppression in patients with advanced ovarian cancer [12]. Moreover, macrophages have been identified as important contributors to ovarian cancer metastasis [9,13]. However, their role in adhesion of ovarian cancer cells to mesothelial cells, a key step in ovarian cancer cell metastasis, remains unclear. In the present study, we examined the potential effects of macrophages on adhesion of ovarian cancer cells to mesothelial cells and the underlying molecular mechanisms.

## 2. Materials and Methods

### 2.1. Materials

M199 medium, Rowell Park Memorial Institute (RPMI) 1640 medium, fetal bovine serum (FBS), penicillin, and streptomycin were purchased from Life Technologies, Inc. (Grand Island, NY, USA). The RNA extraction kit was purchased from Intron Biotechnology (Seoul, Republic of Korea). FMN1, ITGA2, COL13A1, VEGFC, NRG1, and β-actin oligonucleotide primers and ITGA2 and VEGFC siRNA were purchased from Bioneer Technology (Seoul, Republic of Korea). 2-mercaptoethanol and phorbol myristate acetate (PMA) were purchased from Sigma-Aldrich Corp. (St. Louis, MO, USA). CCR2 antagonist RS102895 was obtained from Santa Cruz Biotechnology (Santa Cruz, CA, USA). CCR5 antagonist fuscin (3,4,7,9-Tetrahydroxy-6-methyl-1H-phenalen-1-one) was purchased from Adipogen Life Sciences (San Diego, CA, USA). JNK inhibitor SP600125, PI3K/Akt inhibitor LY294002, and JAK2/STAT3 inhibitor AG490 were obtained from Calbiochem (San Diego, CA, USA). Dimethyl sulfoxide (DMSO) and CellTracker^TM^ were obtained from Invitrogen (Grand Island, NY, USA). Recombinant human CC chemokine ligand 2 (CCL2) and CCL5 were purchased from Thermo Fisher Scientific (Waltham, MA, USA).

### 2.2. Cell Culture and Conditioned Medium

The human mesothelial cell line Met5A, human monocyte cell line THP-1, and human ovarian cancer cell lines A2780 and SKOV3 were originally obtained from the American Type Culture Collection (Manassas, VA, USA). The immortalized ovarian surface epithelial IOSE80PC cell lines were generated by transfection with SV40-T antigen of ovarian surface epithelial cells and provided from N. Auersperg (University of British Columbia, Vancouver, BC, Canada) and A. Godwin (Fox Chase Cancer Center, Philadelphia, PA, USA). Met5A cells were cultured in M199 medium supplemented with 10% FBS, penicillin (100 U/mL), and streptomycin sulfate (100 μg/mL). A2780, SKOV3, and IOSE80PC cells were cultured in RPMI 1640 medium supplemented with 5% FBS, penicillin (100 U/mL), and streptomycin sulfate (100 μg/mL). THP-1 cells were cultured in RPMI 1640 medium supplemented 5% FBS, 1% penicillin (100 U/mL), streptomycin sulfate (100 μg/mL), and 0.05 mM 2-mercaptoethanol at 37 °C in a humidified atmosphere (95% air/5% CO_2_). THP-1 cells were treated with PMA (100 nM) for 24 h to induce macrophage differentiation.

Cell adhesion was used to verify THP-1 cell differentiation. To obtain the conditioned medium (CM), cells (1.0 × 10^5^ cells/mL) were seeded in 100 mm culture dish in 10 mL complete medium for 24 h, and the medium was collected from these cultures. The medium was centrifuged for 3 min at 2500 rpm, and the supernatants (i.e., CM) were collected and kept in storage at −80 °C until use. THP-1 macrophage, A2780, SKOV3, and IOSE80PC-stimulated mesothelial cells (M-Met5A, A-Met5A, S-Met5A, and I-Met5A cells, respectively) were prepared by stimulating the Met5A cells for 24 h with the CM of THP-1 macrophages, A2780, SKOV3, and IOSE80PC cells, respectively

### 2.3. Adhesion Assay

In 96-well plates, Met5A cells were grown until confluence. Trypsinization was used to separate ovarian cancer cells, followed by washing with PBS and a 45 min incubation with CellTracker^TM^ probe at 37 °C. RPMI 1640 medium with 0.1% FBS was used to wash CellTracker^TM^-labeled cells to remove the excess dye before adding them to Met5A cells (4.0 × 10^4^ cells/well for A2780 cells and 1.0 × 10^4^ cells/well for SKOV3 cells). After 30 min incubation at 37 °C, the non-adherent cells were gently washed off, the fluorescence in each well was imaged, and the fluorescence was measured in pixels using the Scion Image Software (Scion Corp, Frederick, MD, USA). To perform inhibition studies, Met5A cells were exposed to each inhibitor 1 h before the cell adhesion assay.

### 2.4. RNA Isolation and Real-Time RT-PCR

The cellular RNA was extracted following the manufacturer’s instructions using the Easy Blue^®^ kits (Intron Biotechnology, Seoul, Republic of Korea). Total RNA was reverse transcribed using first-strand cDNA synthesis kit (Amersham Pharmacia Biotech, Oakville, ON, Canada). Real-time PCR was performed using Thermal Cycler Dice Real Time PCR System (Takara, Tokyo, Japan). The following primers were utilized for SYBR green real-time RT-PCR: for FMN1 sense primer, 5′-AGC TTT GCT CAA ATT GAC GC-3′ and antisense primer 5′-TCC CTT CGG TTC AGT TTT GG-3′; for ITGA2, sense primer, 5′-AGG AAC ATG GGA ACT GGA GG-3 and anti-sense primer, 5′-GGC TGA GTT GCA GGT GAG AA-3′; for COL13A1, sense primer, 5′-CGG TAA ACC AGG AGA CAT GG-3′ and anti-sense primer, 5′-GAA CCT CTG CTC CTG GAT TG-3′; for VEGFC, sense primer, 5′-CAG CTA AGG AAA GGA GGC TG-3′ and anti-sense primer, 5′-CCA AAC TCC TTC CCC ACA TC-3′; for NRG1, sense primer, 5′-GGA ATG GCC GAT GTG TAT GT-3′ and anti-sense primer, 5′-GAA ATG GGT GAG AGA GCC TG-3; for β-actin, sense primer, 5′-CAA ACA TGA TCT GGG TCA TC-3′ and anti-sense primer, 5′-GCT CGT CGT CGA CAA CGG CT-3′. A dissociation curve analysis showed a single peak. From three independent observations, the mean cycle threshold (Ct) of the target gene was determined and normalized using the mean Ct of the β-actin, an internal control gene.

### 2.5. Gene Knockdown Using Small Interfering RNA

Small interfering RNAs (siRNAs) for ITGA2 and VEGFC, and negative control siRNA were synthesized using Bioneer technology. Using Lipofectamine (Invitrogen), siRNA was transfected into the cells at a final dose of 100 nM, as suggested by the manufacturer. Briefly, the cells were plated in 6-well culture plates and incubated for 24 h for adherence and proliferation before being transfected with siRNA. Each transfection mixture was prepared by mixing siRNA and Lipofectamine in serum-free Opti-MEM and incubating the mixture for 15 min at room temperature. The transfection mixture was gently added to the cells, which were allowed to recover for an additional 24 h before further experimental treatments.

### 2.6. mRNA Sequencing

Global transcript profiling was performed in Met5A cells stimulated with CM from macrophages (M-Met5A), IOSE80PC (I-Met5A), and ovarian cancer cells (A-Met5A and S-Met5A). QuantSeq 3 mRNA-Seq was performed by eBiogen, Inc. (Seoul, Republic of Korea). Briefly, the QuantSeq 3′ mRNA-Seq Library Prep Kit (Lexogen, Vienna, Austria) was used to generate an Illumina sequencing library with 500 ng of RNA. QuantSeq 3 mRNA-Seq reads were aligned using Bowtie2 [14]. Based on counts from unique and multiple alignments, differentially expressed genes were identified using coverage in Bedtools [15]. GenowizTM version 4.0.5.6 (Ocimum Biosolutions, Hyderabad, India) was used to process the RT (read count) data using the global normalization protocol. The DAVID (http://david.abcc.ncifcrf.gov/, accessed on 30 January 2017) [16] and Medline (http://www.ncbi.nlm.nih.gov/, accessed on 30 February 2017) databases were used to classify the genes. A fold change of 2 was applied in this study, and a log2 normalized read count of ≥4 was applied to minimize false counts. The Kyoto Encyclopedia of Genes and Genomes (KEGG) (http://www.kegg.jp/, accessed on 22 May 2017) pathway database, which links genomic data to functional data, was used to systematically analyze gene functions.

### 2.7. Cytokine Antibody Arrays

Human cytokine antibody array kits C-series 3 were purchased from Ray Biotech Inc. (Norcross, GA, USA). Using antibody-based arrays, the concentrations of cytokines and chemokines in CM generated from different cell types were measured. Experiments were performed following the manufacturer’s recommendations. Cytokine array membranes were blocked before being treated with 1 mL of CM. After washing, membranes were mixed with 2 mL of diluted biotin-conjugated antibodies. The mixture was then gently shaken for 2 h. As a final step, the membranes were incubated with horseradish peroxidase (HRP)-conjugated streptavidin. Chemiluminescence detection system and Image Quant LAS-4000 (Fujifilm Life science) were used to identify the presence of cytokines or chemokines.

### 2.8. Statistical Analysis

Statistics are shown as the mean ± SD of three independent experiments that were carried out in triplicate. GraphPad Prism version 5 (GraphPad, San Diego, CA, USA) was used to calculate statistically significant differences using one-way analysis of variance (ANOVA) or the Student’s *t*-test. Statistical significance was defined as a *p*-value of less than 0.05.

## 3. Results

### 3.1. Ovarian Cancer Cells Showed an Increased Adhesion to Macrophage-Stimulated Mesothelial Cells

**Figure 1:**
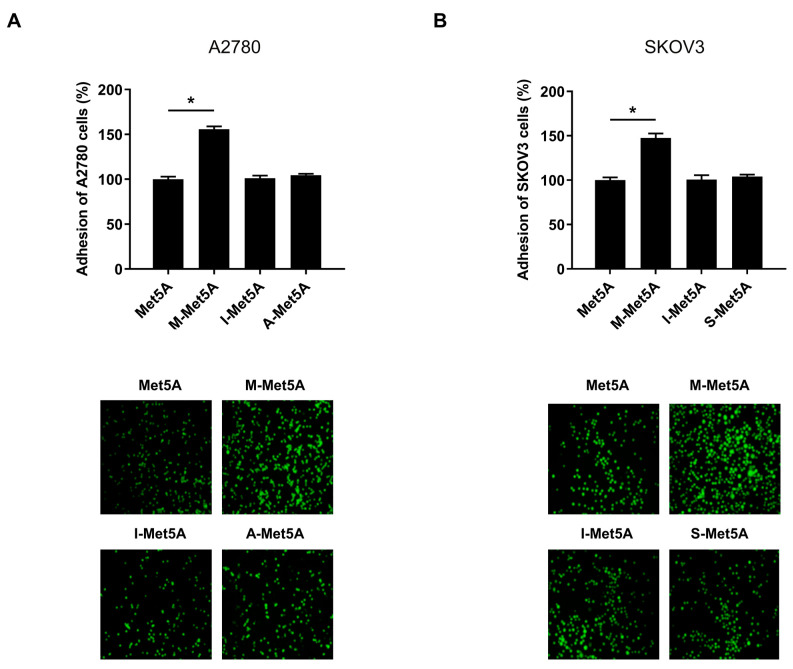
Effect of macrophages on adhesion of ovarian cancer cells to mesothelial cells. Adhesion assays were performed to investigate the adhesion of ovarian cancer cells to the human mesothelial Met5A cells. A2780 (**A**) and SKOV3 (**B**) cells were stained with CellTracker^TM^ and cultured on Met5A, M-Met5A, I-Met5A, A-Met5A or S-Met5A cell layers in 96-well plates for 30 min at 37 °C. The total fluorescence in each well was analyzed using fluorescence microphotography. M-Met5A, Met5A cells stimulated by the conditioned medium (CM) of macrophages; I-Met5A, Met5A cells stimulated by CM from immortalized ovarian surface epithelial (IOSE80PC) cells; A-Met5A, Met5A cells stimulated by the CM of A2780 ovarian cancer cells; S-Met5A, Met5A cells stimulated by the CM of SKOV3 ovarian cancer cells. * *p* < 0.05.

Accumulating evidence suggests that macrophages are critical mediators of tumor progression and metastasis. We investigated whether macrophages, one of the key non-tumor cells in ovarian tumor microenvironments, could affect the adhesion of ovarian cancer cells to mesothelial cells. Human mesothelial Met5A cells were stimulated by conditioned medium (CM) of macrophages, ovarian cancer cells (A2780 and SKOV3), and immortalized ovarian surface epithelial cells (IOSE80PC). Adhesion of A2780 and SKOV3 cells to macrophage-stimulated mesothelial cells (M-Met5A) was approximately 1.5-fold higher than that of unstimulated Met5A cells (Figure 1A,B). However, the adhesion of ovarian cancer cells to A-Met5A cells (Met5A cells stimulated by the CM of A2780 ovarian cancer cells) and S-Met5A cells (Met5A cells stimulated by the CM of SKOV3 ovarian cancer cells) was similar to that of I-Met5A cells (Met5A cells stimulated by the CM of IOSE80PC cells) and unstimulated Met5A cells, indicating that ovarian cancer cells were unable to induce adhesion of Met5A cells as compared to macrophages. Collectively, these results suggested that macrophages, but not cancer cells, can stimulate mesothelial cells to promote ovarian cancer cell adhesion.

### 3.2. Enhanced Expression of Adhesion-Related Genes in Macrophage-Stimulated Mesothelial Cells

**Figure 2:**
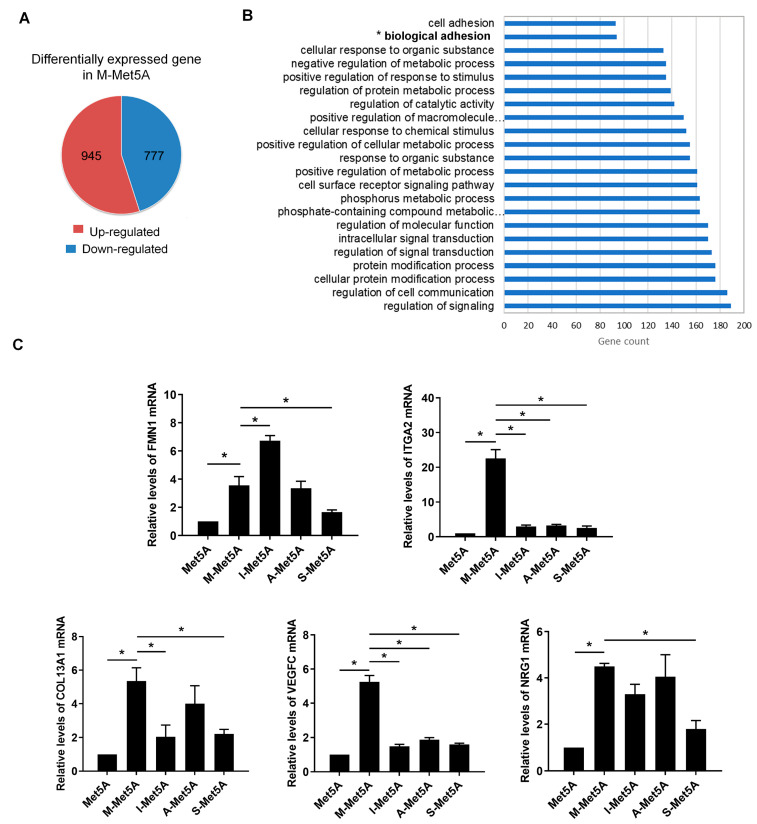
Effect of macrophages on expression of adhesion-related genes in mesothelial cells. (**A**) mRNA sequencing analysis was performed to identify differentially expressed genes (DEGs) in M-Met5A cells (A fold change of ≥2, log2 normalized read count of ≥4 to minimize false counts). (**B**) Functional analysis for the differentially expressed genes in macrophage-stimulated mesothelial cells. The up-regulated 945 genes in M-Met5A cells were classified by DAVID analysis. The figure shows the number and percentage of significantly modulated genes that fit into several ontology function groups, including biological adhesion (94 genes) and cell adhesion (93 genes). (**C**) Real-time RT-PCR shows the expression of five selected genes (*FMN1*, *ITGA2*, *COL13A1*, *VEGFC*, and *NRG1*) in control, M-Met5A, I-Met5A, A-Met5A, and S-Met5A cells. * *p* < 0.05.

To identify differentially expressed adhesion-related genes in M-Met5A cells, we performed global transcript profiling using mRNA sequencing. There were obvious differences in gene expression between M-Met5A and control Met5A cells, showing 1722 differentially expressed genes (DEGs) with at least a 2-fold increase or decrease (Figure 2A). DAVID analysis revealed 94 adhesion-related genes that were classified into the biological adhesion functional category and were upregulated in M-Met5A cells (Figure 2B and Supplementary Table S1). To identify adhesion-related genes specifically increased in mesothelial cells stimulated by macrophages, we chose five genes from the panel of 94 genes that showed more than a 4-fold increase in M-Met5A cells and less than a 2-fold increase in A-Met5A, S-Met5A, and I-Met5A cells compared to control Met5A cells (Supplementary Table S2). These genes (*FMN1*, *ITGA2*, *COL13A1*, *VEGFC*, and *NRG1*) were further validated by real-time RT-PCR (Figure 2C). All five genes were upregulated in M-Met5A cells compared to control Met5A cells. However, only ITGA2 and VEGFC were expressed at significantly lower levels in A-Met5A, S-Met5A, and I-Met5A cells compared to M-Met5A cells. Overall, these observations suggested that the expression of the adhesion-related genes *ITGA2* and *VEGFC* may be associated with the increased adhesiveness of M-Met5A cells toward ovarian cancer cells.

### 3.3. Increased Expression of ITGA2 and VEGFC in Macrophage-Stimulated Mesothelial Cells Promotes Ovarian Cancer-Mesothelial Cell Adhesion

**Figure 3:**
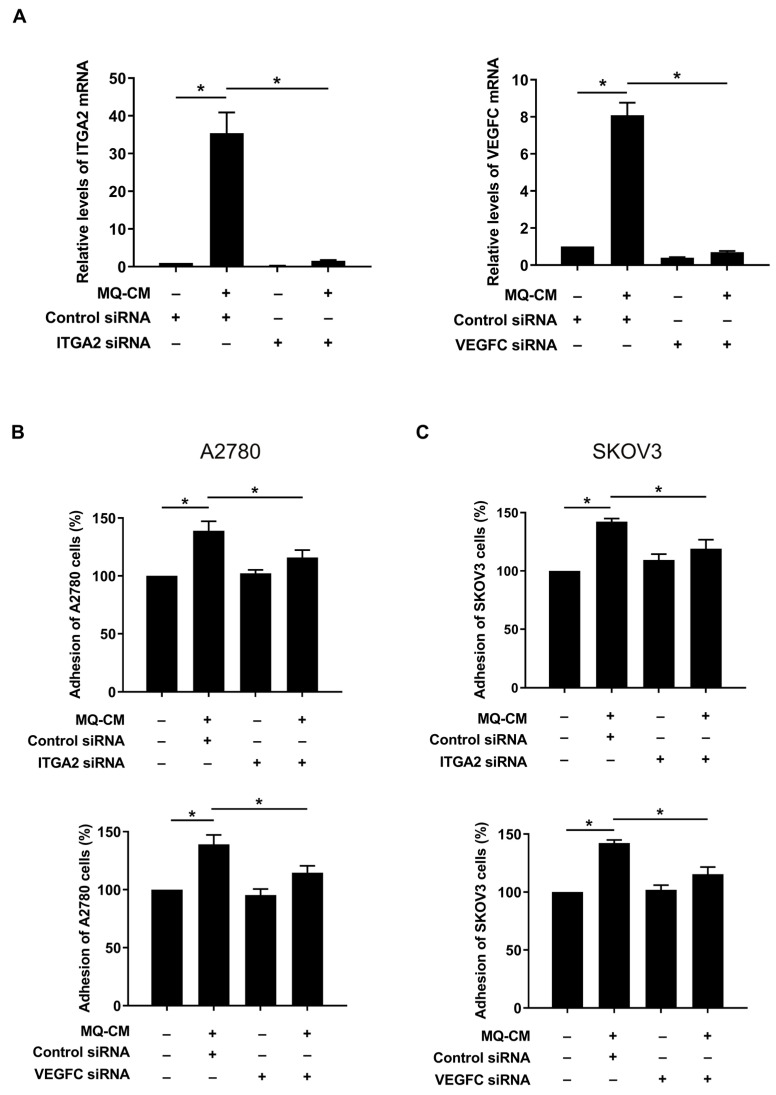
Effect of ITGA2 and VEGFC on adhesion of ovarian cancer cells to mesothelial cells. Met5A cells were transfected with ITGA2 and VEGFC siRNA (100 nM) or control siRNA (100 nM) following addition of conditioned medium (CM) from macrophages (MQ). (**A**) Real-time RT-PCR was performed to measure ITGA2 and VEGFC mRNA levels in Met5A cells. (**B**,**C**) Adhesion assays were performed to investigate the adhesion of ovarian cancer and Met5A cells. A2780 (**B**) and SKOV3 (**C**) cells, after incubation with CellTracker^TM^, were co-incubated on Met5A cells in 96-well plates for 30 min. * *p* < 0.05.

Previous studies have shown that ITGA2 and VEGFC play important roles in the invasion and angiogenesis of various cancers, including gastric and ovarian cancer [17,18]. To determine whether ITGA2 and VEGFC are involved in the enhanced adhesiveness of M-Met5A cells, we further investigated the effect of ITGA2 and VEGFC knockdown on ovarian cancer mesothelial cell adhesion. Transfection with ITGA2 and VEGFC siRNAs significantly suppressed the mRNA expression of ITGA2 and VEGFC in M-Met5A cells (Figure 3A). Knockdown of ITGA2 and VEGFC in M-Met5A cells significantly inhibited the adhesion of A2780 (Figure 3B) and SKOV3 cells (Figure 3C) to M-Met5A cells. These findings imply that increased expression of mesothelial ITGA2 and VEGFC by macrophages are associated with enhanced ovarian cancer-mesothelial cell adhesion.

### 3.4. JNK and Akt Pathways in Macrophage-Stimulated Mesothelial Cells Were Associated with Enhanced Ovarian Cancer-Mesothelial Cell Adhesion

**Figure 4:**
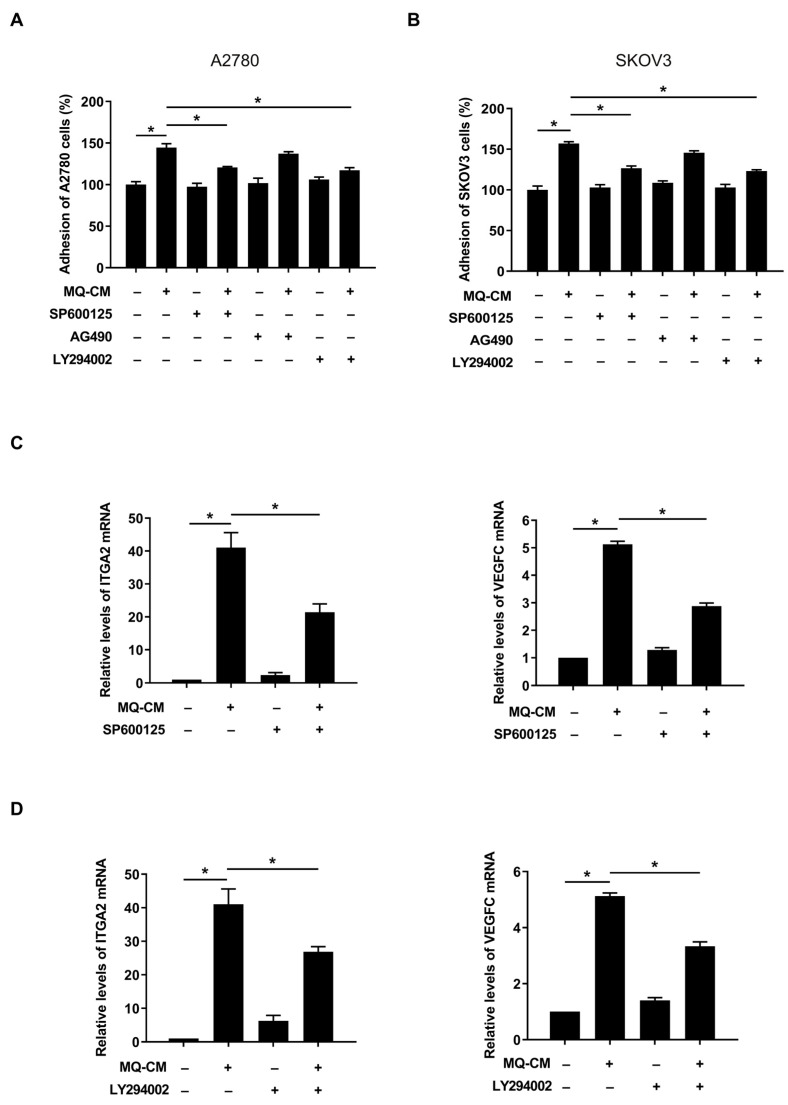
Involvement of JNK, STAT3, and Akt in adhesion of ovarian cancer cells to mesothelial cells. After pretreatment with JNK inhibitor SP600125 (20 μM), STAT3 inhibitor AG490 (30 μM), and Akt inhibitor LY294002 (20 μM), Met5A cells were stimulated by the conditioned medium (CM) of macrophages (MQ-CM). (**A**,**B**) Adhesion assays were performed to investigate the adhesion of ovarian cancer and Met5A cells. A2780 (**A**) and SKOV3 (**B**) cells were incubated with CellTracker^TM^ followed by co-incubation with Met5A cells in 96-well plates for 30 min. (**C**,**D**) Real-time RT-PCR was performed to measure ITGA2 and VEGFC mRNA levels in Met5A cells. * *p* < 0.05.

To investigate the underlying molecular mechanisms by which macrophages promote the upregulation of adhesion-related genes in mesothelial cells, we first examined the common transcription factors that regulate ITGA2 and VEGFC using the UCSC Human Genome Browser Gateway (https://genome.ucsc.edu/cgi-bin/hgGateway) [19]. We identified three common transcription factors cJUN, cFOS, and STAT3 (Supplementary Table S3). The c-Jun N-terminal kinase (JNK) pathway can potentiate activator protein-1 (AP-1) composed of c-JUN and c-FOS by phosphorylation of c-JUN [20]. Additionally, according to the KEGG pathway enrichment analysis, ITGA2 and VEGFC were involved in the Akt pathway. Therefore, we investigated the involvement of the JNK, STAT3, and Akt pathways in the enhanced adhesiveness of macrophage-stimulated mesothelial cells. Western blot analysis showed that the JNK inhibitor SP600125 (20 μM), the STAT3 inhibitor AG490 (30 μM), and Akt inhibitor LY294002 (20 μM) substantially inhibited the activation of the JNK, STAT3, and Akt pathway, respectively (Supplementary Figure S1). It has been demonstrated that SP600125 and LY294002 significantly decreased ovarian cancer cell adhesion to M-Met5A cells; however, the STAT3 inhibitor AG490 did not (Figure 4A,B). Furthermore, the JNK and Akt inhibitors considerably reduced the ITGA2 and VEGFC mRNA levels in M-Met5A cells (Figure 4C,D). Taken together, these data indicated that the JNK and Akt pathways regulate the mRNA expression of ITGA2 and VEGFC, thereby modulating the adhesiveness of M-Met5A cells toward ovarian cancer cells.

### 3.5. CCL2 and CCL5 Secreted by Macrophages Are Associated with Enhanced Ovarian Cancer-Mesothelial Cell Adhesion

**Figure 5:**
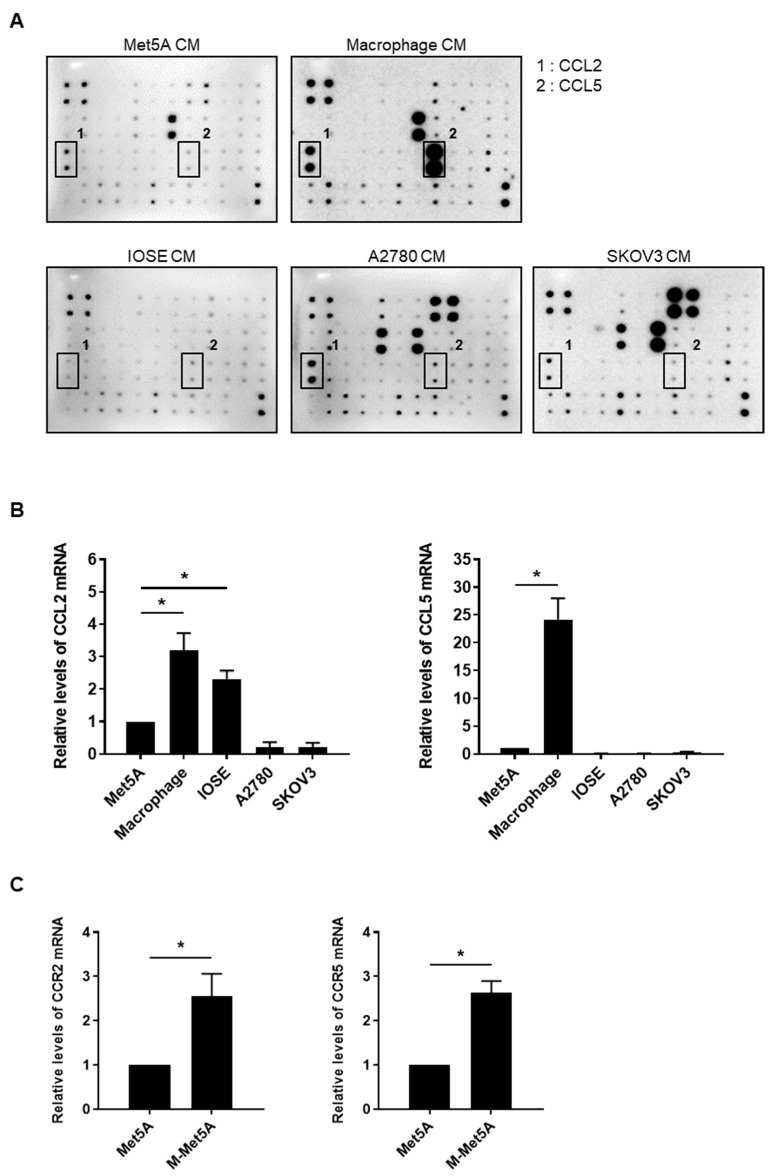
Production of CCL2 and CCL5 by macrophages and enhanced expression of CCR2 and CCR5 in macrophage-stimulated mesothelial cells (**A**) A cytokine array was used to analyze the cytokine/chemokine profiles of the conditioned medium (CM) of Met5A, macrophage, IOSE, A2780, and SKOV3 cells. (**B**) Real-time RT-PCR was performed to measure the mRNA levels of CCL2 and CCL5 in Met5A, macrophages, IOSE, A2780, and SKOV3 cells. (**C**) The mRNA levels of CCR2 and CCR5 were evaluated and quantified in Met5A and M-Met5A cells by real-time RT-PCR. * *p* < 0.05.

**Figure 6:**
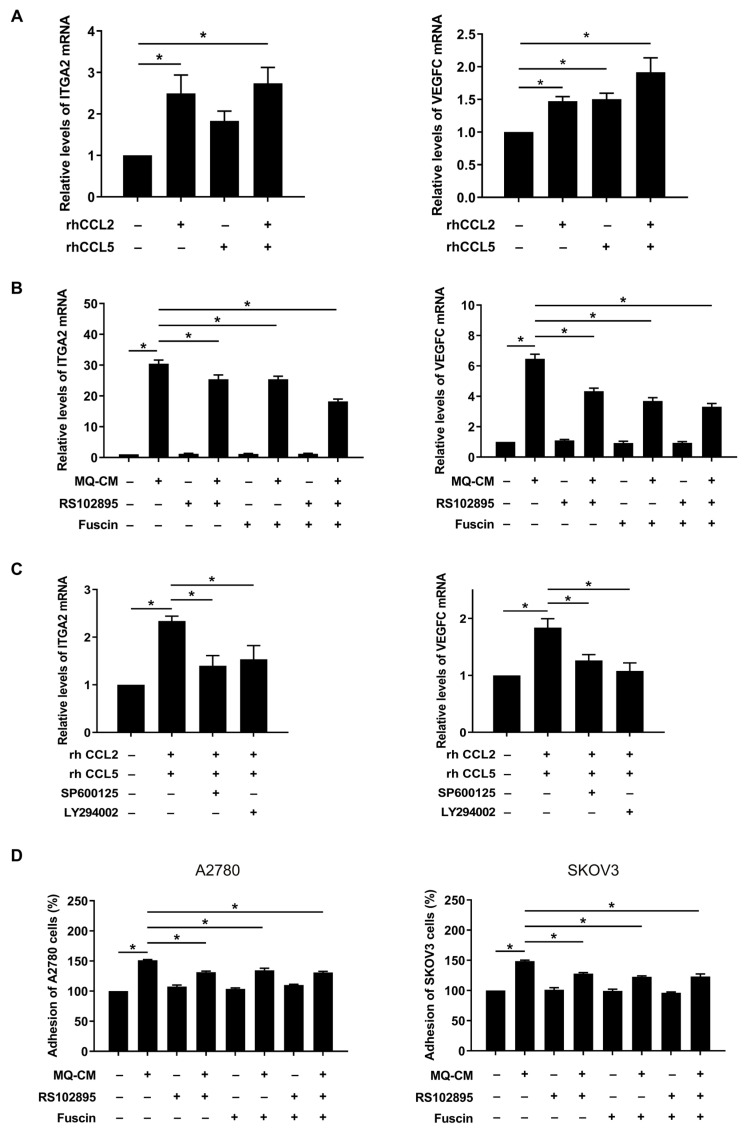
Involvement of CCL2 and CCL5 in adhesion of ovarian cancer cells toward mesothelial cells. (**A**–**C**) Real-time RT-PCR was performed to measure the mRNA levels of ITGA2 and VEGFC in Met5A cells. (**A**) Met5A cells were treated with recombinant human CCL2 (rhCCL2; 200 ng/mL) and/or CCL5 (rhCCL5; 200 ng/mL). (**B**) After pretreatment with CCR2 antagonist RS102895 (10 μM) and/or CCR5 antagonist fuscin (5 μM), Met5A cells were stimulated by macrophage CM (MQ-CM). (**C**) After pretreatment with JNK inhibitor SP600125 (20 μM) or Akt inhibitor LY294002 (20 μM), Met5A cells were co-treated with rhCCL2 (200 ng/mL) and rhCCL5 (200 ng/mL) for 24 h. (**D**) Adhesion assay was performed to investigate the adhesion of ovarian cancer cells and Met5A cells. Met5A cells were stimulated with MQ-CM following pretreatment with CCR2 antagonist RS102895 (10 μM) and/or CCR5 antagonist fuscin (5 μM). A2780 and SKOV3 cells, after incubation with CellTracker^TM^, were further co-incubated with Met5A cells in 96-well plates for 30 min. * *p* < 0.05.

To determine which soluble factors that are present in the CM of macrophages stimulated the expression of adhesion-related genes in mesothelial cells, a human cytokine antibody array was used to elucidate the cytokine profile of the macrophages. As shown in Figure 5A, macrophages produced higher amounts of CCL2 and CCL5 compared to Met5A, ovarian cancer, and IOSE80PC cells. Accordingly, overexpression of CCL2 and CCL5 in macrophages was confirmed (Figure 5B). Moreover, their receptors, CC chemokine receptor type 2 (CCR2) or type 5 (CCR5), were found to be upregulated in M-Met5A cells compared to untreated macrophages (Figure 5C). CCL2 [21,22] and CCL5 [23,24], in addition to acting as chemokines, are known to regulate cancer progression by regulating various signaling pathways of cancer cells in the tumor microenvironment. Therefore, we examined whether these two cytokines secreted by macrophages could enhance mesothelial cell adhesion ability by regulating the expression of cell adhesion-related genes in mesothelial cells. Treatment with recombinant human CCL2 (rhCCL2) and/or CCL5 (rhCCL5) induced mRNA expression of ITGA2 and/or VEGFC in Met5A cells (Figure 6A). In addition, the CCR2 antagonist RS102895 and/or the CCR5 antagonist fuscin considerably reduced ITGA2 and VEGFC mRNA expression in M-Met5A cells (Figure 6B). Furthermore, we found that JNK and Akt inhibitors significantly reduced ITGA2 and VEGFC expression in Met5A cells costimulated with rhCCL2 and rhCCL5 (Figure 6C). Moreover, the ability of ovarian cancer cells to adhere to M-Met5A cells was decreased by CCR2 and/or CCR5 antagonists (Figure 6D). Taken together, these data indicated that CCL2 and CCL5 secreted by macrophages can increase the expression of adhesion-related genes ITGA2 and VEGFC via the JNK and Akt pathways in Met5A cells, resulting in enhanced ovarian cancer-mesothelial cell adhesion.

## 4. Discussion

The mesothelium, a single layer of mesothelial cells that covers the peritoneal cavity, plays a major role in the spread of ovarian cancer since it acts as a site for tumor cell attachment, implantation, and growth [25]. Additionally, mesothelial cells have been shown to actively mediate cancer metastasis [26]. However, the mechanism by which normal mesothelial cells become metastasis-associated mesothelial cells remains largely unknown. Some reports have shown the effects of cancer cells on mesothelial cell-stimulated ovarian cancer progression. For example, mesothelial cells enhance endothelial migration and tube formation by promoting vascular endothelial growth factor (VEGF) production in the presence of ovarian cancer cells [27]. Kenny et al. suggested that TGF-β secreted from ovarian cancer cells increased fibronectin expression in mesothelial cells, which is associated with adhesion, invasion, proliferation, and metastasis of ovarian cancer cells [28]. However, in this study, the ovarian cancer cell adhesion of mesothelial cells stimulated by the cancer cells was similar to that of control mesothelial cells, suggesting that ovarian cancer cells are unable to promote the adhesion activity of mesothelial cells. Instead, we found that macrophage-stimulated mesothelial cells adhered more efficiently to ovarian cancer cells. To the best of our knowledge, this is the first study to demonstrate the role of macrophages in the adhesion of ovarian cancer cells to mesothelial cells. Considering that the adhesion is a key step in ovarian cancer cell metastasis, macrophages in the abdominal cavity may stimulate the ovarian cancer metastasis. In fact, this is consistent with other studies suggesting a potential role of macrophages in ovarian cancer metastasis [9,29]. 

TAMs have been shown to secrete soluble factors that interact with various cells in the tumor microenvironment, including cancer cells [30]. For example, a study suggested that transforming growth factor beta-induced (TGFBI) protein and tenascin C secreted by macrophages promote the progression of ovarian cancer [31]. Intriguingly, we identified the chemotactic factors CCL2 and CCL5 in the CM of macrophages and found that they were associated with the adhesion of ovarian cancer cells to mesothelial cells. CCL2 and CCL5 have been suggested to act as important mediators between tumor and host cells in the tumor microenvironment [21,23,32]. For example, CCL2 secreted by TAMs increases chemoresistance in breast cancer cells [33]. In addition, CCL5 derived from TAMs has been reported to increase the stemness and metastatic capacity of prostate cancer cells [24]. There are also some reports regarding the potential roles of CCL2 and CCL5 in ovarian cancer. We demonstrated that high levels of CCL2 in ovarian cancer are associated with paclitaxel resistance via autocrine signaling and macrophage recruitment [34]. Moreover, CCL2 secreted by mesothelial cells has been suggested to promote peritoneal metastasis and adherence of ovarian cancer cells [35]. Mesothelial CCR5 stimulated by macrophage-derived factors was also found to be associated with mesothelial-ovarian cancer cell adhesion [36]. In this study, we demonstrated that CCL2 and CCL5 derived from macrophages induced mesothelial expression of the adhesion-related genes *ITGA2* and *VEGFC* to increase the adhesion of ovarian cancer cells. To the best of our knowledge, this is the first report to demonstrate the effects of CCL2 and CCL5 on mesothelial cells. Taken together, these results suggest that CCL2/5 can originate from noncancerous cells such as macrophages in the ovarian cancer microenvironment, as well as tumor cells, and may play a role in ovarian cancer progression and chemoresistance. 

The molecular and cellular mechanisms underlying the ovarian cancer cell adhesion towards the mesothelial cells remain poorly understood [28,36]. Here, we found that the mesothelial expression of ITGA2 and VEGFC was induced by macrophages and enhanced the adhesion of ovarian cancer cells to mesothelial cells. To the best of our knowledge, this is the first study to demonstrate the potential roles of mesothelial ITCA2 and VEGFC in cancer-mesothelial cell adhesion. Integrins are members of a family of cell surface receptors that mediate cell–cell and cell–extracellular matrix (ECM) interactions [37]. The integrin family has been suggested to play an important role in cell adhesion and tumor progression in ovarian cancer metastasis [38]. Integrin α2 (ITGA2), an important collagen receptor, is associated with focal adhesion and extracellular matrix-receptor interactions [39]. Notably, a recent study demonstrated that ITGA2 promotes peritoneal metastasis of ovarian cancer through collagen-mediated cell adhesion and focal adhesion signaling [40]. Vascular endothelial growth factor (VEGF) is a pro-angiogenic cytokine that enhances the proliferation and migration of endothelial cells and the permeability of existing vessels [41]. Although VEGFC, a lymphangiogenic VEGF, has been demonstrated to induce the metastasis and progression of various types of cancers [18,42,43], there are few reports showing its direct role in intercellular adhesion. VEGFs are known to increase the expression of adhesion-related genes in human endothelial cells [44]. In addition, VEGFC acts as a signaling ligand for integrin α9β1 [45] that can stimulate the focal adhesion of melanoma cells [46]. Based on these findings, we speculate that mesothelial ITGA2 may directly increase the adhesive capability of mesothelial cells through collagen-mediated adhesion and that VEGFC may contribute to adhesion by acting as an inducer of adhesion-related gene expression and signaling pathways in both mesothelial and cancer cells. However, the exact mechanism of action of these adhesion-related genes in mesothelial cells requires further elucidation.

We found that the upregulated expression of ITGA2 and VEGFC in M-Met5A was closely related to activation of the JNK and Akt signaling pathways. The JNK pathway is a mitogen-activated protein kinase (MAPK) pathway that promotes the expression of related genes by activating the AP-1 transcription factor composed of cJUN and cFOS. According to recent research, the JNK pathway may play a significant role in mediating the spread and advancement of human malignancies, including ovarian cancer [47,48]. Interestingly, AP-1 promotes the expression of integrin α2β1 and VEGF in leukemia [49] and endothelial cells [50], respectively. These data suggested that JNK pathway activation in macrophage-affected mesothelial cells may promote the expression of *ITGA2* and *VEGFC*, thereby increasing the adhesion of mesothelial cells to ovarian cancer cells. The phosphatidylinositol 3-kinase (PI3K)/Akt pathway is mainly known as a key regulator of survival during cellular stress [51]. In addition, this signaling pathway promotes angiogenesis and metastasis of cancer cells through a variety of mechanisms [52,53]. Various genetic alterations that induce the activation of Akt signaling have been observed in ovarian cancer [54]. Chemokine ligands, including CCL2 and CCL5, are secreted by various cell types and are widely associated with cell signaling via interactions with cell surface receptors (CCRs) [55]. Notably, the CCL2/CCR2 and CCL5/CCR5 axes have been shown to activate the JNK and Akt pathway [56,57]. In fact, we found that CCR2 antagonist RS102895 and CCR5 antagonist fuscin markedly inhibited the activation of JNK and Akt in human mesothelial Met5A cells (Supplementary Figure S2). These data are consistent with our present findings, which demonstrated that CCL2 and CCL5 secreted from macrophages can stimulate their receptors on mesothelial cells to activate the JNK and Akt pathways, thereby promoting *ITGA2* and *VEGFC* expression. 

Our data demonstrate for the first time that macrophages play a crucial role in the adhesion of ovarian cancer cells to mesothelial cells, a key step in ovarian cancer cell metastasis, by inducing the mesothelial expression of adhesion-related genes. ITGA2, VEGFC, and their regulatory pathways, JNK and Akt, play critical roles in increasing the adhesion of macrophage-stimulated mesothelial cells to ovarian cancer cells. CCL2 and CCL5, soluble factors secreted by macrophages, are involved in macrophage-mediated mesothelial changes. In this regard, it can be speculated that some therapies targeting the adhesion-related genes and/or signaling in the macrophages and mesothelial cells can effectively control ovarian cancer metastasis. In fact, several monoclonal antibodies targeting TAM-associated molecules are currently under development and have been shown to inhibit ovarian cancer progression [58]. Although further studies including in vivo experiments are needed, the macrophage-derived CCL2/5-mesothelium axis could be a potential target for treating metastatic ovarian cancer. Our findings thereby highlight the importance of the potential effects of noncancerous cells on ovarian cancer progression.

## Data Availability

The datasets generated during and/or analyzed during the current study are available from the corresponding author on reasonable request.

## Supplementary Materials

The following supporting information can be downloaded at: https://www.mdpi.com/article/10.3390/cells12030384/s1, Table S1: List of 94 adhesion-related genes; Table S2: Five adhesion-related genes (*FMN1*, *ITGA2*, *COL13A1*, *VEGFC*, and *NRG1*) were selected by a gene selection criteria; Table S3: List of transcription regulators conserved in transcription factor binding site of adhesion-related genes; Figure S1: Effect of RS102895 and fuscin on the activation of JNK and Akt in human mesothelial cells; Figure S2: Effect of SP600125, AG490, and LY294002 on the activation of JNK, STAT3, and Akt in human mesothelial cells.
