# Supplementary material for: Macrophages Promote Ovarian Cancer-Mesothelial Cell Adhesion by Upregulation of ITGA2 and VEGFC in Mesothelial Cells
Source: Cells. 2023 Jan 20;12(3):384. doi: 10.3390/cells12030384 (PMC9913165; doi:10.3390/cells12030384)
Supplement: Supplementary file 1 [file cells-12-00384-s001.zip › cells-2160909-supplementary.pdf]

Supplementary Table S1. List of 94 adhesion-related genes. The table show the 94 genes in “biological adhesion” class and fold-change present in our 3’ mRNA sequencing analysis.

No	Gene symbol	Gene description	List ID	Fold change
1	ICAM4	intercellular adhesion molecule 4	NM_001544	34.39
2	EPHA4	EPH receptor A4	NM_004438	24.21
3	SLAMF7	SLAM family member 7	NM_021181	23.64
4	ACTN2	actinin alpha 2	NM_001103	20.94
5	ARHGAP6	Rho GTPase activating protein 6	NM_013427	19.11
6	LRRC15	leucine rich repeat containing 15	NM_130830	18.86
7	CLDN18	claudin 18	NM_016369	17.98
8	NLRP3	NLR family pyrin domain containing 3	NM_004895	17.07
9	PAK6	p21 (RAC1) activated kinase 6	NM_020168	17.06
10	FMN1	formin 1	NM_001277313	10.61
11	WNT7B	Wnt family member 7B	NM_058238	9.47
12	ITGA2	integrin subunit alpha 2	NM_002203	8.88
13	COL13A1	collagen type XIII alpha 1 chain	NM_080801	8.35
14	TGM2	transglutaminase 2	NM_004613	7.75
15	AGER	advanced glycosylation end-product specific receptor	NM_001136	7.58
16	LAT	linker for activation of T-cells	NM_014387	7.06
17	SNAI2	snail family transcriptional repressor	NM_003068	6.86
18	DISC1	disrupted in schizophrenia 1	NM_018662	6.38
19	MYB	MYB proto-oncogene, transcription factor	NM_005375	6.31
20	IL1B	interleukin 1 beta	NM_000576	6.14
21	LPXN	leupaxin	NM_004811	6.04
22	VEGFC	vascular endothelial growth factor C	NM_005429	5.10
23	NT5E	5'-nucleotidase ecto	NM_002526	5.03
24	LAMC2	laminin subunit gamma 2	NM_005562	4.88
25	CD34	CD34 molecule	NM_001773	4.65
26	NRG1	neuregulin 1	NM_013956	4.08
27	ARHGAP18	Rho GTPase activating protein 18	NM_033515	4.06
28	CD6	CD6 molecule	NM_006725	3.92
29	SERPINE1	serpin family E member 1	NM_000602	3.84
30	TPBG	trophoblast glycoprotein	NM_006670	3.80
31	EGR1	early growth response 1	NM_001964	3.78
32	COL17A1	collagen type XVII alpha 1 chain	NM_000494	3.77
33	ADGRG1	adhesion G protein-coupled receptor G1	NM_005682	3.77
34	EGFLAM	EGF like, fibronectin type III and laminin G domains	NM_152403	3.73
35	AMIGO2	adhesion molecule with Ig like domain 2	NM_181847	3.69
36	SOX9	SRY-box 9	NM_000346	3.69

37	SCARF1	scavenger receptor class F member 1	NM_003693	3.66
38	ITGA6	integrin subunit alpha 6	NM_000210	3.66
39	COL8A1	collagen type VIII alpha 1 chain	NM_001850	3.64
40	FAS	Fas cell surface death receptor	NM_000043	3.55
41	AMIGO1	adhesion molecule with Ig like domain 1	NM_020703	3.55
42	ITGB8	integrin subunit beta 8	NM_002214	3.53
43	MYO10	myosin X	NM_012334	3.47
44	PTPRR	protein tyrosine phosphatase, receptor type R	NM_002849	3.45
45	MTSS1	MTSS1, I-BAR domain containing	NM_014751	3.35
46	BCL6	B-cell CLL/lymphoma 6	NM_001706	3.30
47	RGMB	repulsive guidance molecule family member b	NM_001012761	3.13
48	COL6A3	collagen type VI alpha 3 chain	NM_004369	3.11
49	VCAM1	vascular cell adhesion molecule 1	NM_001078	2.96
50	PLAU	plasminogen activator, urokinase	NM_002658	2.88
51	GREM1	gremlin 1, DAN family BMP antagonist	NM_013372	2.85
52	SORBS1	sorbin and SH3 domain containing 1	NM_006434	2.74
53	BTN2A2	butyrophilin subfamily 2 member A2	NM_006995	2.66
54	IL23A	interleukin 23 subunit alpha	NM_016584	2.66
55	COL5A3	collagen type V alpha 3 chain	NM_015719	2.64
56	SMAD7	SMAD family member 7	NM_005904	2.60
57	COL7A1	collagen type VII alpha 1 chain	NM_000094	2.59
58	IL4R	interleukin 4 receptor	NM_000418	2.53
59	CCL28	C-C motif chemokine ligand 28	NM_148672	2.53
60	ITGA11	integrin subunit alpha 11	NM_001004439	2.48
61	LAMA1	laminin subunit alpha 1	NM_005559	2.48
62	TNIP1	TNFAIP3 interacting protein 1	NM_006058	2.44
63	PLXNB1	plexin B1	NM_002673	2.44
64	C1QTNF1	C1q and tumor necrosis factor related protein 1	NM_030968	2.44
65	RND1	Rho family GTPase 1	NM_014470	2.43
66	DOCK5	dedicator of cytokinesis 5	NM_024940	2.42
67	CD58	CD58 molecule	NM_001779	2.39
68	CD44	CD44 molecule (Indian blood group)	NM_000610	2.38
69	PNP	purine nucleoside phosphorylase	NM_000270	2.37
70	VEGFA	vascular endothelial growth factor A	NM_003376	2.32
71	THBS3	thrombospondin 3	NM_007112	2.31
72	NDRG1	N-myc downstream regulated 1	NM_006096	2.30
73	DOCK9	dedicator of cytokinesis 9	NM_015296	2.30
74	KITLG	KIT ligand	NM_003994	2.30
75	MUC1	mucin 1, cell surface associated	NM_002456	2.29
76	SMAGP	small cell adhesion glycoprotein	NM_001031628	2.28
77	RELB	RELB proto-oncogene, NF-kB subunit	NM_006509	2.27
78	ALCAM	activated leukocyte cell adhesion molecule	NM_001627	2.25

79	MINK1	misshapen like kinase 1	NM_015716	2.22
80	ADAM8	ADAM metallopeptidase domain 8	NM_001109	2.20
81	PDZD2	PDZ domain containing 2	NM_178140	2.19
82	COL4A6	collagen type IV alpha 6 chain	NM_001847	2.18
83	MAP3K14	mitogen-activated protein kinase kinase kinase 14	NM_003954	2.17
84	GLMN	glomulin, FKBP associated protein	NM_053274	2.16
85	NUMB	NUMB, endocytic adaptor protein	NM_003744	2.13
86	CD59	CD59 molecule	NM_000611	2.12
87	LAMB3	laminin subunit beta 3	NM_000228	2.12
88	CD274	CD274 molecule	NM_014143	2.10
89	ZFP36L1	ZFP36 ring finger protein like 1	NM_004926	2.09
90	PCDH1	protocadherin 1	NM_032420	2.08
91	ETS1	ETS proto-oncogene 1, transcription factor	NM_001143820	2.07
92	DST	dystonin	NM_001723	2.05
93	ADORA2A	adenosine A2a receptor	NM_000675	2.02
94	PPFIBP1	PPFIA binding protein 1	NM_003622	2.01

Supplementary Table S2. Five adhesion-related genes (*FMN1, ITGA2, COL13A1, VEGFC,* and *NRG1*) were selected by a gene selection criteria. Among the 94 adhesion-related genes, we choose only those genes that showed more than 4-fold increase in M-Met5A cells. Additionally, we excluded genes that were increased more than 2-fold in A-Met5A, S-Met5A, and I-Met5A cells compared with control Met5A cells.

No	Gene symbol	Gene description	List ID	Fold change			
				M-Met5A /Met5A	I-Met5A /Met5A	A-Met5A /Met5A	S-Met5A /Met5A
1	FMN1	formin 1	NM_001277313	10.61	1.01	1.01	1.33
2	ITGA2	integrin subunit alpha 2	NM_002203	8.88	1.67	1.00	1.84
3	COL13A1	collagen type XIII alpha 1 chain	NM_080801	8.35	1.85	1.90	1.89
4	VEGFC	vascular endothelial growth factor C	NM_005429	5.10	1.69	1.58	1.51
5	NRG1	neuregulin 1	NM_013956	4.08	1.74	1.99	1.26

Supplementary Table S3. List of transcription regulators conserved in transcription factor binding site of adhesion-related genes. Each transcription regulators found onto ITAG2 and VEGFC binding regions were listed by UCSC Human Genome Browser Gateway (https://genome.ucsc.edu/cgi-bin/hgGateway accessed on 5 Jun 2017). Among the several transcription regulators of ITGA2 (the number of 81) and VEGFC (the number of 31), cJUN, cFOS, and STAT3 were found onto ITGA2 and VEGFC binding sites.

**Genes**	**Transcription regulators**									
**ITGA2** (#81)	ARID3A	ATF2	ATF3	BATF	BCL11<br>A	BCL3	BHLHE40	BRCA1	CEBPB	CEBPD
	cFOS	CHD2	cJUN	cMYC	CREB1	CTCF	E2F4	ELF1	ELK4	EP300
	ESR1	FAM48A	FOSL1	FOSL2	FOXA1	FOXA2	GABPA	GATA2	GATA3	GTF2F1
	HDAC2	HNF4A	HNF4G	IRF4	JUNB	JUND	KAP1	MAFF	MAFK	MAX
	MAZ	MBD4	MEF2A	MEF2C	MXI1	MYBL2	NFIC	NR3C1	PAX5	POLAR2A
	POLR2A	POU2F2	RAD21	RBBP5	RCOR1	RELA	REST	RFX5	RUNX3	RXRA
	SIN3AK2<br>0	SMARCB<br>1	SMC3	SP1	SPI1	STAT1	STAT3	TAF1	TBP	TCF12
	TCF7L2	TEAD4	TFAP2<br>A	TFAP2<br>C	USF1	USF2	YY1	ZBTB7<br>A	ZNF14<br>3	ZNF217
	ZNF263									
**VEGF<br>C** (#31)	CEBPB	cFOS	cJUN	cMYC	CTCF	E2F1	E2F4	EP300	EZH2	FOSL2
	FOXA1	GATA2	JUND	MAFK	NR3C1	POLR2A	RAD21	RBBP5	REST	SIN3AK2<br>0
	SMC3	SP2	SRF	STAT3	TAF1	TBP	TCF12	TCF7L2	ZBTB3<br>3	ZNF143
	ZNF217									

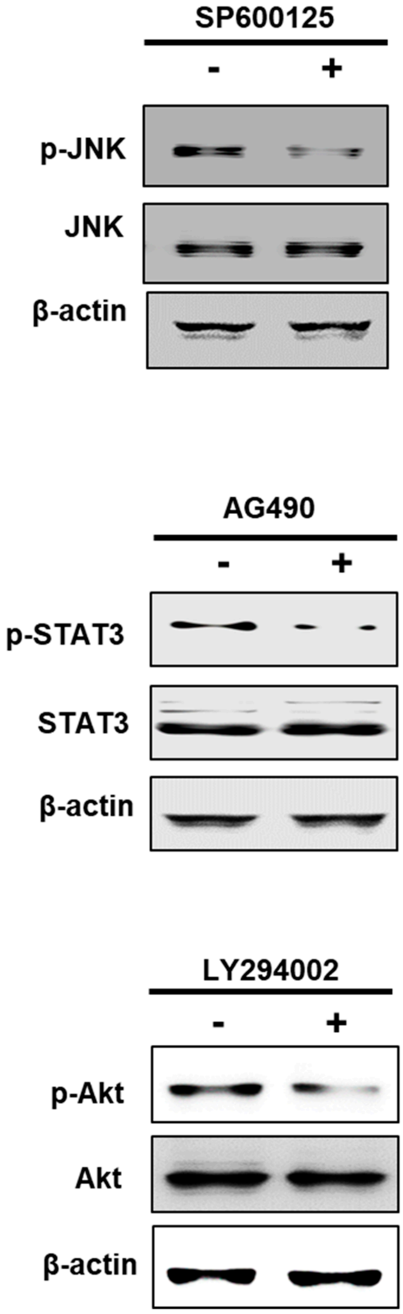

Supplementary Figure S1. Effect of SP600125, AG490, and LY294002 on the activation of JNK, STAT3, and Akt in human mesothelial cells. Met5A cells were treated with JNK inhibitor SP600125 (20 μM), STAT3 inhibitor AG490 (30 μM), and Akt inhibitor LY294002 (20 μM) for 24 h. Western blot analysis was performed to evaluate the activation of JNK, STAT3, and Akt for the treatment with SP600125, AG490, and LY294002, respectively.

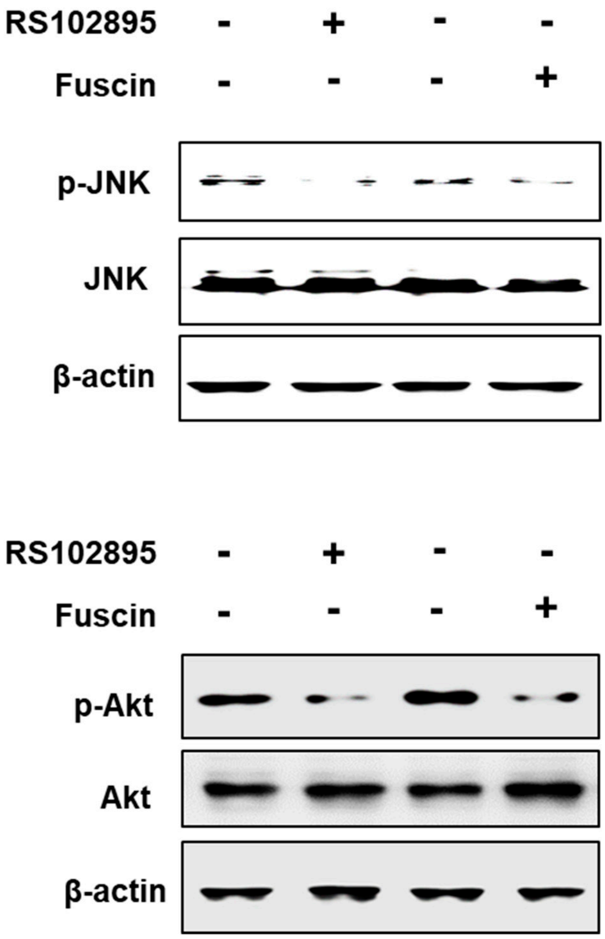

Supplementary Figure S2. Effect of RS102895 and fuscin on the activation of JNK and Akt in human mesothelial cells Met5A cells were treated with CCR2 antagonist RS102895 (10 μM) and CCR5 antagonist fuscin (5 μM) for 24 h. Western blot analysis was performed to evaluate the activation of JNK and Akt.
